# Antioxidant Activity and Chemical Composition of Geranium Oil and Its Synergistic Potential against Pneumococci with Various Antibiotic Combinations

**DOI:** 10.3390/plants12173080

**Published:** 2023-08-28

**Authors:** Berrak Dumlupinar, Gökçe Şeker Karatoprak, Betül Demirci, Esra Küpeli Akkol, Eduardo Sobarzo-Sánchez

**Affiliations:** 1 Department of Biochemistry, Faculty of Pharmacy, İstanbul-Cerrahpaşa University, Istanbul 34116, Türkiye; berrak.dumlupinar@iuc.edu.tr; 2 Department of Pharmacognosy, Faculty of Pharmacy, Erciyes University, Kayseri 38039, Türkiye; gskaratoprak@erciyes.edu.tr; 3 Department of Pharmacognosy, Faculty of Pharmacy, Anadolu University, Eskisehir 26470, Türkiye; 4 Department of Pharmacognosy, Faculty of Pharmacy, Gazi University, Ankara 06330, Türkiye; 5 Instituto de Investigación y Postgrado, Facultad de Ciencias de la Salud, Universidad Central de Chile, Santiago 8370292, Chile; 6 Department of Organic Chemistry, Faculty of Pharmacy, University of Santiago de Compostela, 15782 Santiago de Compostela, Spain

**Keywords:** *Geranium* oil, *S. pneumoniae*, antibiotics, antioxidant, antibacterial, synergism

## Abstract

The essential oil of *Pelargonium graveolens* L. is valuable for its therapeutic benefits, so this study aimed to determine the synergistic effect of the combination of the essential oil of this plant with antibiotics instead of the extracts prepared with various solvents. In addition, the second goal of this study was to determine whether the essential oil combined with various antibiotics increased the overall killing activity in mouse macrophage cells with the aim of introducing an immunotherapeutic approach to the infection treatments used today. Herein, the volatile profile of *Geranium* oil (G.O) was analyzed using GC/MS. The current study sought to assess the synergistic characteristics of several antibiotic combinations using G.O against pneumococci, as well as the oil’s antioxidant and antimicrobial activities. The major components of the oil were citronellol, geraniol, and isomenthone. In the combinations of G.O and antibiotics, the synergism of the *Streptococcus pneumoniae* to antibiotics advanced. When the time-kill data were evaluated, G.O + antibiotic combinations quickly diminished the viable cell count of *S. pneumoniae* from the 6th h. In this study, the combined use of existing antibiotics used in infection treatments with G.O could improve antibiotic effectiveness and thus prevent bacteria from developing antibiotic resistance.

## 1. Introduction

*Streptococcus pneumoniae* is found in the upper respiratory tract microbiota. This bacterium causes infections, such as sinusitis, meningitis, pneumonia, otitis media, and blood inflammation, as well as severe life-threatening infections, such as pneumonia and meningitis [1]. Penicillin, amoxicillin, cefuroxime, and erythromycin are generally preferred in the treatment of pneumococcal infections. However, in recent years, unsuccessful results have been obtained in treatment as a result of the emergence of penicillin-resistant pneumococci. Antibiotics used to treat pneumococcus have been noticed in the literature [2]. The most commonly used treatment methods are beta-lactam antibiotics: ampicillin, penicillin G, cephalosporins (cefoxitin, cefotaxime, ceftriaxone); aminoglycoside class antibiotics: gentamicin and streptomycin; and combinations with bacteriostatically effective chloramphenicol and tetracyclines. However, combining an antibiotic that induces beta-lactamase synthesis, such as cefoxitin with a beta-lactam antibiotic may cause the breakdown of beta-lactam, and the co-administration of two beta-lactam antibiotics may cause inconveniences. In aminoglycoside and cephalosporin combinations used in current infection treatments, aminoglycosides and cephalosporins may inactivate each other by a chemical interaction. Another problem is the possibility of losing the lethal effect of the antibiotic by administering bactericidal and bacteriostatic antibiotics together. The use of combinations of penicillin and chlortetracycline in the treatment of pneumococcal patients resulted in higher mortality than patients treated with penicillin alone [3].

*Pelargonium graveolens* L. (Geraniaceae) is a shrub native to South Africa. Geranium oil (G.O), which has a strong hydrophilic property, contains high levels of acyclic monoterpene alcohols, geraniol, citronellol, and linalool, which provides it with a high bioactivity [4]. G.O is especially useful in stimulating the immune system, sore throats, tonsillitis, asthma, and excessive mucus secretion; health problems, such as fungus, general infections, and acne caused by microorganisms show that it has antioxidant and antimicrobial benefits [5,6].

In our study, the antimicrobial effect against *S. pneumoniae* was determined by combining the bactericidal antibiotics of the beta-lactam derivative penicillin G and ampicillin, and the quinolone antibiotic ciprofloxacin, and the essential oil obtained from *P. graveolens.* The composition of the G.O was determined using gas chromatography–mass spectrometry to effectively and safely develop natural antibiotics for the treatment or alleviation of respiratory tract infections, especially those caused by antibiotic-resistant *S. pneumoniae* strains. In addition, with the determination of whether the combination of the obtained G.O with some antibiotics increases the overall killing activity in mouse macrophage cells, an immunotherapeutic approach to the infection treatments used today was revealed. Our third goal was to determine the antioxidant effects of *P. graveolens* essential oil. The terpenoids, hydrocarbons, alcohol, and aldehydes in the structure of this G.O have high bioactive properties. For this reason, this study analyzed whether the use of G.O in place of extracts formulated with diverse solvents, and the combination of this G.O with antibiotics showed a synergistic effect.

## 2. Results and Discussion

### 2.1. Chemical Analysis of Essential Oil Using Gas Chromatography (GC) and Gas Chromatography–Mass Spectrometry (GC/MS)

Citronellol, geraniol, and their esters were found to be the main compounds; the chemical composition of the G.O is shown in Table 1. The main constituents of the oil were citronellol (41.2%), geraniol (8%), and isomenthone (4.1%). High-quality oil is found in the leaves, stems, and petioles of the plant [7]. High levels of citronellol (~29%), geraniol (~12%), delta-selen (~9%), and citronellil formate (~7%) were observed to be the major components in the Geranium oil obtained from the plant *Pelargonium graveolens* [8]. It is thought that these compounds in the essential oil may be related to the bioactivity of the oil. The high antibacterial and antifungal effects of citronellol and geraniol was demonstrated in previous studies [9,10].

### 2.2. DPPH^●^ Radical Scavenging Effect

It is very important to remove harmful and disease-causing radicals from the body. For this purpose, the DPPH^●^ radical, a stable nitrogen-centered radical, was used to measure the antiradical effects of the samples. The DPPH^●^ radical is a stable radical with a maximum absorbance of 517 nm. When it is reduced to hydrazine derivatives of the DPPH radical using electron and hydrogen atom transfer by antioxidant properties, the absorbance decreases [11].

**Figure 1:**
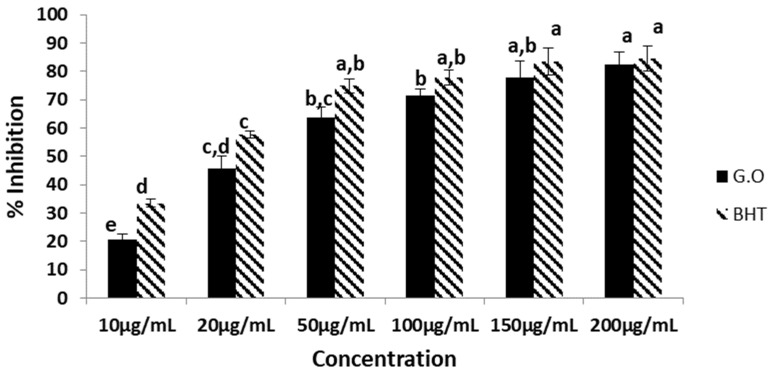
DPPH radical scavenging effect of Geranium oil. The same lowercase letters (a–e) are not significantly (*p* > 0.05) different. (*n* = 3, G.O: Geranium oil, BHT: Butylated Hydroxytoluene).

The G.O scavenges the DPPH^●^ radical depending on the concentration at a physiological pH. As can be seen in Figure 1, an 82.4% inhibition was found at the highest concentration (200 µg/mL) and 20.54% at the lowest concentration (10 µg/mL). BHT, which is used as a standard, was 84.54% inhibition at 200 µg/mL against the radical and exhibited a 33.41% inhibition at 10 µg/mL. In the study, using the aerial parts of *P. endlicherianum*, the inhibitory effect of β-carotene/linoleic acid oxidation and the free radical (DPPH^●^) scavenging effects were examined, and it was seen that the aerial parts of *P. endlicherianum* had strong antioxidant activity [12]. In a different study, the DPPH^●^ scavenging effects of *P. endlicherianum,* with a 70% methanol root extract, showed a 0.23 mg/mL IC50 value [13]. The aqueous extract and essential oil of *P. graveolens* showed that the IC50 values ranged from 0.19 to 0.05 mg/mL (stems), 0.39 to 0.04 mg/mL (leaves) for hydrosols; and from 63.70 to 1.56 mg/mL (leaves) and 64.88 to 1.12 mg/mL (stems) for essential oils. The potential use of essential oils as a good source of antioxidants was expressed [14].

### 2.3. Determination of β-Carotene/Linoleic Acid Oxidation Inhibitory Effect

**Figure 2:**
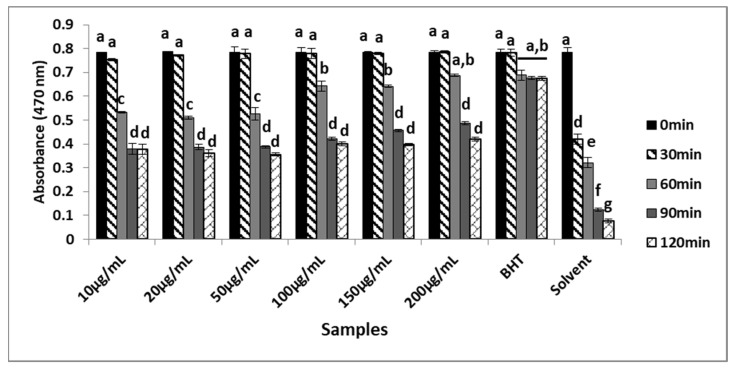
Inhibitory effect of Geranium oil on β-carotene/linoleic acid oxidation. The same lowercase letters (a–g) are not significantly (*p* > 0.05) different. Data are expressed as mean ± standard deviation, *n* = 3.

Lipids in cell membranes are rich in linoleic acid and arachidonic acid, which are unsaturated fatty acids that are fairly susceptible to oxidation [15,16]. Therefore, it is important to use experimental environments for the oxidation of lipids while screening the antioxidant effects of the G.O analyzed in this study. Peroxyl free radicals consist of a hydrogen reduction in the linoleic acid and oxidation of unsaturated β-carotene. The β-carotene-linoleic acid withering test is utilized to detect these peroxide free radicals. Antioxidant structures in the components of the G.O decrease the oxidation of β-carotene by hydro peroxide. If the antioxidant capacity of the Geranium oil is high, the rate of degradation of β-carotene decreases [17]. The oxidation inhibitory effect of G.O was investigated over time and a time-dependent change was observed. As seen in Figure 2, while BHT showed the highest activity, G.O at a 200 µg/mL concentration prevented oxidation in the first 60 min, almost at the same rate as BHT. Samples prepared at 100, 150, and 200 µg/mL concentrations were found to be more active at 60, 90, and 120 min. In the determination of the radical scavenging effects and the β-carotene/linoleic acid oxidation inhibitory effects of the leaves of *P. radula* (Cav.) L’Hérit species with ethanol, water, and essential oil extracts, it was observed that the extracts were found to be antioxidants, while the essential oil was more active than the extracts [18]. In another study, *P. endlicherianum* with a 70% methanol extract and its butanol and ethyl acetate fractions were found to inhibit oxidation to a statistically equivalent degree with gallic acid [13].

### 2.4. ABTS^●+^ Radical Scavenging

**Figure 3:**
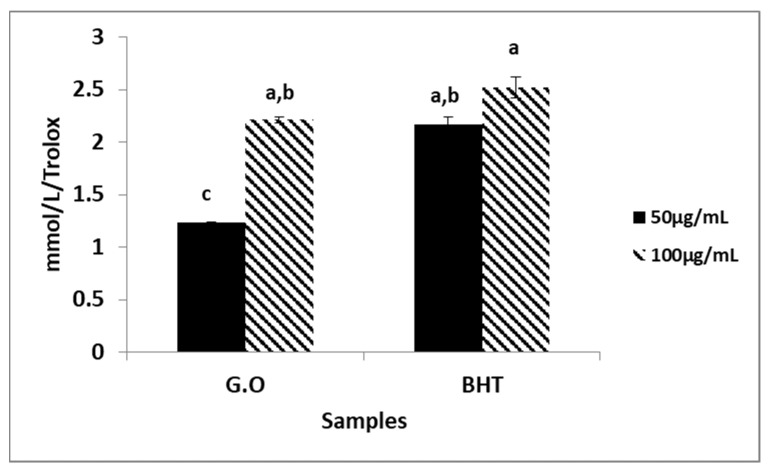
ABTS^●+^ radical scavenging effect of Geranium oil. (Data are expressed as mean ± standard deviation, *n* = 3, G.O: Geranium oil, BHT: Butylated Hydroxytoluene). The same lowercase letters (a–c) are not significantly (*p* > 0.05) different.

The ABTS^●+^/TEAC method, one of the most widely used antioxidant methods, depends on the reduction in the absorbance of the ABTS^●+^ radical, which shows the characteristic wavelength absorption spectrum with a maximum at 660, 734, and 820 nm, in the presence of an antioxidant [19]. In this study, the ABTS^●+^ radical scavenging potentials of G.O and BHT were investigated at doses of 50 and 100 µg/mL. The G.O showed the highest activity at a 100 µg/mL dose. The G.O did not show as much activity as the BHT, which was used as a positive standard (Figure 3). To prove the radical scavenging effects of the essential oil, the ABTS^+●^ radical scavenging effect test was also determined, and the results are given in Figure 3 as equivalent to Trolox. The essential oil was analyzed at two different doses. The results were found to be 1.21 mmol/L Trolox at 50 µg/mL, 2.21 mmol/L Trolox at 100 µg/mL, and 2.17 mmol/L Trolox for BHT at 50 µg/mL. It was determined that the BHT was more active than the essential oil in both of the doses studied. Methanol and water extracts obtained from the leaves and flowers of *P. graveolens* were found to be comparable to BHT in the DPPH^●^ and ABTS^●+^ radical scavenging effect, while its *Geranium* oil was determined to be more active than BHT [20].

### 2.5. Determination of Cytotoxic Effect in Raw 264.7 Cells

**Figure 4:**
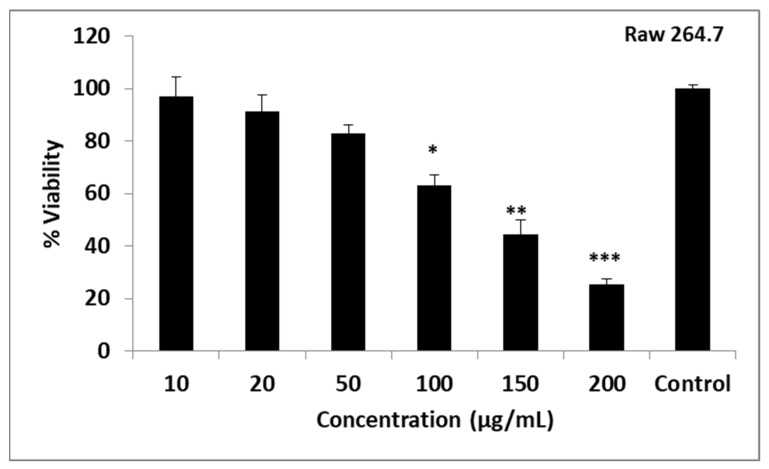
Cytotoxicity of the Geranium oil was assessed using MTT reduction assay against the Raw 264.7 cell line. Data are expressed as mean ± standard deviation, *n* = 3. * *p* < 0.05, ** *p* < 0.01, *** *p* < 0.001.

The non-toxic dose was determined with cytotoxicity studies using Raw 264.7 cells. Viability in the Raw 264.7 cell line was 25.19% at 200 µg/mL concentration; 44.56% at 150 µg/mL concentration; 62.94% at 100 µg/mL concentration; 83.04% at 50 µg/mL concentration; it was found to be 91.21% at 20 µg/mL concentration; and 97.12% at 10 µg/mL concentration. Viability remained below 50% at 200 and 150 µg/mL concentrations. The highest percentage of viability was found at 10 µg/mL concentration (Figure 4). The viability decreased in the concentration range of 10–200 µg/mL, inversely with the increase in concentration.

### 2.6. Minimum Inhibitory Concentration (MIC)

*S. pneumoniae* is susceptible to penicillin, ampicillin, and ciprofloxacin (0.5 mg/L, 0.5 mg/L, and 0.03 mg/L, respectively). In particular, ciprofloxacin was the antibiotic with the lowest MIC. The G.O MIC value was determined to be 10 mg/mL for *S. pneumoniae.*

The MIC value of G.O against *S. pneumoniae* was 10 mg/mL, and the zone diameter was 11 ± 0.5 mm. The MIC value of ciprofloxacin was detected as 0.031 µg/mL with a zone value of 9 ± 0.5 mm. When ciprofloxacin was used in combination with the G.O, the MIC value was 0.015 µg/mL for the antibiotic in the combination and 5 mg/mL for the G.O. The MIC zone diameter of the ciprofloxacin + G.O combination was detected as 8 ± 0.5 mm (Table 2). When penicillin was used in combination with the G.O, the MIC value was found to be 0.125 µg/mL, and the MIC zone diameter was 11 ± 0.5 mm (Table 3).

The MIC value of ampicillin was detected as 0.5 µg/mL with a zone value of 12 ± 1 mm. When ampicillin was used in combination with the G.O, the MIC value was found to be 0.25 µg/mL, and the MIC zone diameter was 11 ± 1 mm (Table 4). It was determined that the antibacterial activity of the combination of ciprofloxacin + G.O, penicillin + G.O, and ampicillin + G.O is higher than when used alone. A synergistic effect was found on *S. pneumoniae*. It was reported in previous studies that essential oils show potent antimicrobial activity on Gram-positive bacteria compared to Gram-negative bacteria by increasing the acidity level, thereby easily infiltrating the bacterial cell membrane thanks to the lipophilic polyphenols in their content [21,22,23].

The results of our in vitro study show that the combined use of the bactericidal quinolone group antibiotic ciprofloxacin, the beta-lactam group antibiotic penicillin, and ampicillin with G.O positively affected the control of the *S. pneumoniae* pathogen, which is known to be the most common cause of upper respiratory system infections.

### 2.7. Time-Kill Assay

**Figure 5:**
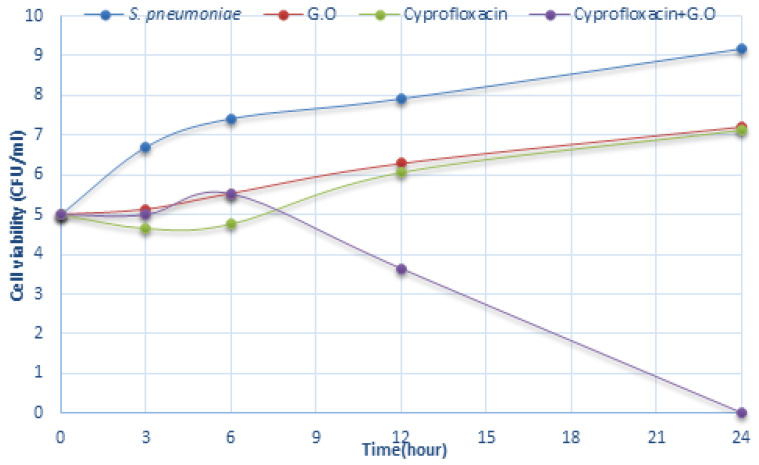
Time-kill analysis of Geranium oil, ciprofloxacin, and a combination of both against *S. pneumoniae*. (*n* = 3, G.O: *Geranium* oil).

**Figure 6:**
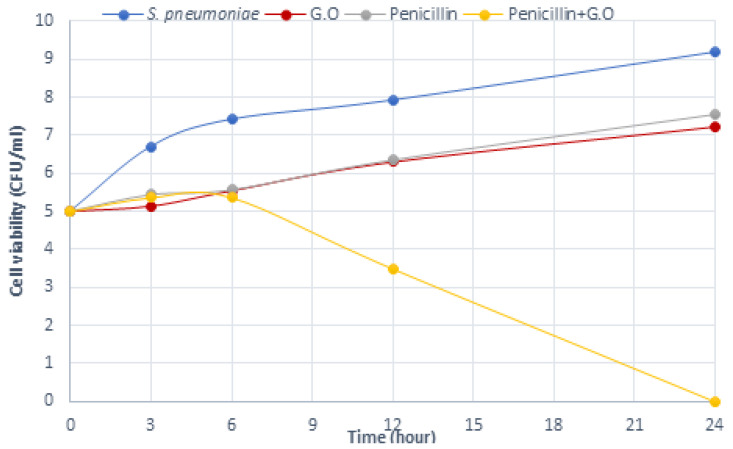
Time-kill analysis of Geranium oil, penicillin, and a combination of both against *S. pneumoniae.* (*n* = 3, G.O: *Geranium* oil).

**Figure 7:**
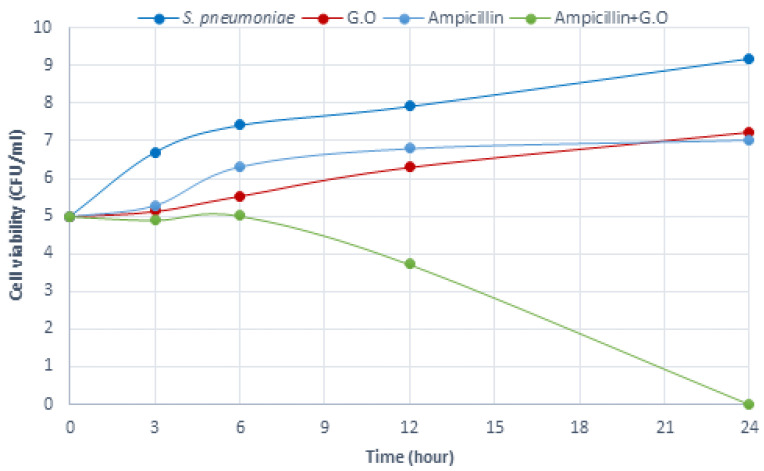
Time-kill analysis of Geranium oil, ampicillin, and combination of both against *S. pneumoniae* (*n* = 3, G.O: *Geranium* oil).

The data for time-dependent killing indicated that there is a synergistic effect between the G.O and antibiotics, and, therefore, more effective than treatments with G.O and/or antibiotics alone. For *S. pneumoniae*, G.O + antibiotic combinations caused a significant rapid reduction in the viable cell count from the 6th h (Figure 5, Figure 6 and Figure 7). Here, the inhibitory effects of ciprofloxacin, penicillin, and ampicillin antibiotics, which are preferred in the treatment of *S. pneumoniae* infections when used together with G.O, against this bacterium were investigated. The mechanism of action of the ciprofloxacin, penicillin, and ampicillin antibiotics used in this study together with G.O caused an increase in the effectiveness of these antibiotics.

### 2.8. Activation of Macrophage Cells

The overall killing ability of the *P. graveolens* oil (G.O) in mouse macrophage cells was examined. Data on the potentiation for antibacterial activity of macrophage cells, detected using antibiotics, are shown in the tables (Table 5 and Table 6). Antibiotic and antibiotic + G.O treatment critically increased the overall killing activity of the macrophage cells compared to the control values (*p* ˂ 0.022). The G.O was more influential than ciprofloxacin and the ciprofloxacin + G.O combination (*p* = 0.001). The logarithmic growth of *S. pneumoniae* exhibited a synergistic effect with the combination of ciprofloxacin + G.O at 4 h, and the number of bacterial cells diminished by about 4 logs. The logarithmic growth of *S. pneumoniae* exposed to the ampicillin + G.O combination at 2 h was inhibited by the macrophage cells (*p* ˂ 0.005). From 2 h, the number of viable cells diminished by almost 3 logs with the synergistic effect of the ampicillin + G.O combination compared to the ampicillin treatment group. It increased the overall killing activity in the macrophage cells in combination with penicillin + G.O against *S. pneumoniae* from the 4th h (*p* ˂ 0.001). Penicillin treatment from 4 h diminished the logarithmic growth of *S. pneumoniae* cells by almost 3 logs, while penicillin + G.O treatment demonstrated a synergistic effect, decreasing the number of viable cells by just about 4 logs. All the antibiotics studied showed synergistic effects with the G.O. The overall killing effect of the *P. endlicherianum* essential oil on leukocyte cells was determined, and the combined use of ampicillin, penicillin, and ciprofloxacin antibiotics with the *Pelargonium* essential oil on *N. meningitidis* and *H. influenza* bacteria advanced the overall killing activity of the leukocyte cells, indicating a synergistic effect [24]. In this study, it was determined that the G.O acquired from this species, combined with antibiotics, had a bactericidal effect against *S. pneumoniae* showing a synergistic effect and raised the overall killing activity of the macrophage cells. For this reason, due to the fact that the components of the G.O are richer in alcohol and aldehydes, it showed a much higher antioxidant and antimicrobial effect.

Although some essential oils do not show any significant inhibitory effects when used alone, they are synergistic. When used in combination with antibiotics, the combination activity exceeds its own potential and indicates an enhanced bactericidal activity [25]. The ratio of multi-resistant bacteria is rising, and this situation is becoming a global problem. As resistance to antibiotics increases, the value of infection control increases. Antibiotic + G.O may be a method that can be used in the treatment of infections.

A combination therapy of antibiotics with various molecules is available in the treatment of some diseases caused by infections. However, when unsuitable antibiotic combinations are used, combined antibiotic treatment not only goes over budget but also has unfavorable side effects and falls short of expectations in terms of therapeutic efficacy. On the other hand, when a side effect persists due to combined antibiotic treatment, it is difficult to attribute it to a particular antibiotic; for this reason, interruption of all antibiotics causes the patient to be subjected to a prolonged duration of treatment, thereby increasing the cost [26]. This is time-consuming as it not only delays the patient’s treatment but it is also expensive. Today, the most important problem in pneumococcal infection is the emergence of penicillin-resistant and multi-resistant strains and their global spread. Other antibiotics, such as erythromycin or norfloxacin are usually used for patients allergic to penicillin. About 5% of all *S. pneumoniae* strains are relatively penicillin-resistant [27]. In recent years, strains of pneumococci have been found to be resistant to erythromycin and fluoroquinolones [28].

## 3. Materials and Methods

### 3.1. GC-FID and GC/MS Analysis

*Geranium* oil (G.O), Chinese (Sigma, St. Louis, MI, USA, W250813) commercial oil, was used in our study. The GC–MS analysis was performed using an Agilent 5975 GC-MSD system. An innowax FSC column (60 m × 0.25 mm, 0.25 µm film thickness) was used with helium as the carrier gas (0.8 mL/min). The GC oven temperature was kept at 60 °C for 10 min and programmed to 220 °C at a rate of 4 °C/min and kept at a constant temperature of 220 °C for 10 min, and then programmed to 240 °C at a rate of 1 °C/min. The Split ratio was adjusted at 40:1. The injector temperature was set at 250 °C. The mass spectra were recorded at 70 eV. The mass range was from m/z 35 to 450. The GC analysis was carried out using an Agilent 6890N GC system. The FID detector temperature was 300 °C. To obtain the same elution order with GC–MS, a simultaneous auto-injection was performed on a duplicate of the same column, applying the same operational conditions. The relative percentage amounts of the separated compounds were calculated using FID chromatograms. The analysis results are given in Table 1. The identification of the essential oil components was carried out by a comparison of their relative retention times with those from authentic samples or by a comparison of their relative retention index (RRI) to a series of n-alkanes. Computer matching against commercial (Wiley GC/MS Library, MassFinder Software 4.0) [29,30] and an in-house “Başer Library of Essential Oil Constituents”, built up by genuine compounds and components of known oils.

### 3.2. Determination of 1,1-Diphenyl-2-picrylhydrazil (DPPH^●^) Radical Scavenging Effect

The DPPH^●^ radical scavenging effects of the essential oil were carried out according to the method of Gyamfi et al. (1999) [31]. The samples were prepared ranging between 10 and 200 µg/mL concentrations and were combined with DPPH solution prepared with 0.1 mM of methanol and Tris-HCl buffer (50 nM, pH 7.4). The essential oil was diluted in 70% ethanol for the antioxidant activity assays. A reagent mixture without the essential oil was used, and the positive control was Butylated Hydroxytoluene (BHT). After 30 min of incubation at 25 °C in the dark, absorbances were evaluated at 517 nm. The percent inhibition values were calculated using the following equation:

Inhibition % = [(Abs_control_ – Abs_sample_)/Abs_control_] × 100 (1)

### 3.3. Determination of β-Carotene Inhibitory Effect

The antioxidant activity of the essential oil was determined according to the β-carotene withering experiment [32]. Then, 25 mL of chloroform was used to dissolve 5 mg of β-carotene. To the vial containing linoleic acid (40 mg) and Tween 20 (400 mg), an aliquot of the β-carotene solution was added. After the chloroform had evaporated, 50 mL of distilled water was slowly added. The positive control was BHT. The control and sample blanks were made without β-carotene. Then, these samples in the tubes were kept in a water bath at 50 °C for 120 min for autoxidation and the degree of fading was monitored by measuring every 15 min. Measurements were taken using a spectrophotometer. At 470 nm, the β-carotene inhibitory effect was calculated according to Oomah and Mazza (1996) [32].

### 3.4. Determination of 2,2′-Azino-Bis (3-Ethylbenzathiazolin-6-Sulfonic Acid) (ABTS^●+^) Radical Scavenging Effect

The ABTS^●+^ radical was formed with an aqueous solution of ABTS (7 mM) and potassium persulfate (K_2_S_2_O_8_) (2.45 mM, final concentration) by keeping it in the dark for 12–16 h, and its absorbance at 734 nm was adjusted to 0.700 (±0.020). The samples were prepared at two different concentrations (50 and 100 µg/mL). The prepared radical solution and the samples were mixed to obtain 990 µL in increments of 10 µL. For a total of 30 min, reaction kinetics were monitored at 734 nm once each minute. The percentages of inhibition measured against concentration were calculated as Trolox equivalent (TEAC) [33].

### 3.5. Raw 264.7 Mouse Macrophage Cell Line and Culture

In this experimental system in vitro, Raw 264.7 mouse macrophage cells were used. The cells were routinely maintained and cultured in Dulbecco’s Modified Eagle Medium (DMEM) from Sigma-Aldrich, supplemented with 10% Foetal Bovine Serum (FBS) from Biowest, 1% penicillin–streptomycin (Sigma-Aldrich), 1% L-glutamine, and 3.7 g/L sodium bicarbonate under a humidified atmosphere (5% CO_2_) at 37 °C *S. pneumoniae*, incubated overnight at 37 °C with BHA medium were suspended in BHB medium. The bacterial suspension was incubated for 2 h at 37 °C in a shaker oven according to the MIC values of the antibiotic/G.O/antibiotic + G.O combinations. The antibiotics/G.O/G.O + antibiotic combination were removed from the tubes after the incubation. The bacterial count was first adjusted to 5 × 10^7^ cfu/mL using a McFarland 0.5 turbidity in BHB and then diluted one-half with the same medium. For the bacteria in a non-antibiotic BHB medium, the above procedures were performed in the same way and this prepared bacterial suspension was included in the control series [34].

### 3.6. Determination of Cytotoxic Effect by MTT Method

The Raw 264.7 mouse macrophage cells were used as the in vitro cell model. In the determination of the cytotoxic effect, the metabolic activities of the cells were determined using MTT (3-(4,5)-dimethylthiazol-2-yl)-2,5-diphenyl tetrazolium bromide). The absorbance was read at a 570 nm wavelength in an ELISA (Agilent BioTek Epoch Microplate Spectrophotometer, USA). The measured value was correlated with the number of living cells [35].

### 3.7. Preparation of Essential Oil Dilutions

After 320 mg of pure essential oil was dissolved in 1280 mL of DMSO, 400 mL of the essential oil solution that was dissolved in the DMSO was taken from it to make a concentration of 80 mg/mL and 1000 mL was completed with medium containing 0.5% Tween 80. Other concentrations were equally reduced (80, 40, 20, 10.5, 2.5, 1.25, 0.62, 0.31, 0.15, 0.07, and 0.03) [36].

### 3.8. Bacterial Culture

*S. penumoniae* ATCC 49619 was obtained from the American Type Culture Collection (ATCC). For disk diffusion, MHA with 5% sheep blood was used (CLSI). Growth of the studied bacteria was achieved by incubating using the Brain Heart Infusion Agar/Broth (BHA/BHB) medium for 20–24 h under anaerobic conditions (5% CO_2_, 35 ± 2 °C) in accordance with CLSI recommendations [37]. The colonies obtained from the cultures were adjusted to 0.5 McFarland standard (1 × 108 cfu/mL) using a physiological saline solution (0.9% NaCl).

### 3.9. Minimum Inhibitory Concentration (MIC)

Since the spectrophotometer values of the wells containing the essential oil did not give a standard result in the measurement range, no antimicrobial effect could be detected in the MIC studies using the microdilution method, and these steps of the study were continued using the agar dilution technique. The initial antibiotic concentrations were diluted according to the EUCAST clinical breakpoint table by selecting the two upper concentrations [38].

### 3.10. Agar Well Diffusion

Antibiotics were dissolved in distilled water, the essential oil was dissolved in DMSO and 0.5% Tween 80, and the concentrations used in this study were prepared. Microorganism suspensions were prepared using 24 h agar cultures. Bacterial suspensions were washed twice with 0.9% physiological saline adjusted to the McFarland standard turbidity at a concentration of 10^8^ cfu/mL. Wells, 6 mm in diameter, were opened using a sterile punch at regular intervals on solidified agar medium, and the antibiotics were dissolved in distilled water, the essential oil was dissolved in DMSO, and the antibiotic + essential oil combinations were added. Meropenem (10 µg/mL) was used as a positive control and solvents (DMSO, Tween 80, and distilled water) were used as negative controls. All of the petri dishes were incubated for 24 h at 37 °C 5% CO_2_ for the growth of bacteria, and the growth inhibition zones were measured in millimeters at the end of this period. The experiment was performed in triplicates. The MIC values of the antibiotics and essential oil were determined using the agar well diffusion method and antibiotic + essential oil combinations were prepared with these values, starting above 2 MIC values [37,38]. The synergistic effect was analyzed according to the fractional inhibitory concentration (FIC) index [34]. FIC of G.O = G.O’s MIC value in the presence of antibiotic/G.O’s MIC value, FIC of antibiotic = Antibiotic’s MIC value in the presence of G.O/Antibiotic’s MIC value, FIC ≤ 0.5 *synergistic*, 0.5 < FIC < 1 *partially synergistic*, FIC = 1 *additive*, 1 < FIC ≤ 4 ineffective, and FIC > 4 antagonistic.

### 3.11. Time-Kill Method

The time-kill method dynamically revealed the bactericidal potential of the G.O based on time and essential oil concentration. The live bacteria counts were made at different times for the time-dependent reduction. Using 5 mL of the Brain Heart Broth (BHB) medium, containing the antibiotics and essential oil, and the antibiotic + essential oil combinations at two lower and two upper concentrations of the MIC value, were separately prepared. Initially, 10^5^ cfu/mL bacteria were used in the time-kill method. After applying the method, as described in previous studies, the control tube and the tube containing the antibiotic/essential oil/antibiotic + essential oils were inoculated with bacteria, the sampling was conducted at 0, 12, and 24 h, after which the colonies were counted [36,39,40].

### 3.12. Effects of Essential Oil and Antibiotic Combinations on Macrophage Cell Functions

The McFarland 0.5 (approximately 1 × 10^8^ CFU/mL) was taken from the bacterial suspension, and the essential oil + antibiotic combinations were incubated. After incubation, the antibiotics, and their combinations were removed from the tubes. The Raw 264.7 mouse macrophage cells (2 × 10^7^ cells/mL) were added into the suspension with a bacterial suspension containing approximately 4 × 10^7^ bacteria cells and allowed to incubate. At 0, 2, 4, 8, and 12 h, the samples were taken from the tubes and vortexed to induce macrophage explosion, and by diluting in the appropriate ratio the previously prepared BHA medium was inoculated onto the surface and after a 24 h incubation at 37 °C the colonies were counted. The number of bacteria killed by the macrophages was determined by comparing the values found with the control values. This experiment was carried out in triplicate [41,42,43].

### 3.13. Statistical Evaluation Method

The results were evaluated using the Windows SPSS 13.0 program using the one-way analysis of variance (One-Way ANOVA) and appropriate post-hoc tests (Tukey and Dunnet T3).

## 4. Conclusions

In the present study, the chemical composition and the biological activities of *Geranium* oil was investigated in detail. According to the results, *Geranium* oil has the ability to scavenge DPPH radicals in a concentration range of 20 to200 µg/mL with the same significance as BHT. At the same time, it was found to be as effective as BHT in scavenging ABTS radicals at a concentration of 100 µg/mL. The G.O at a 200 µg/mL concentration prevented oxidation in the first 60 min, almost the same rate as BHT. In the analysis made using GC–MS, the main components of the oil were determined to be citronellol, geraniol, and isomenthon.

The potential of using *P. graveolens* essential oil together with antibiotics as a new treatment method against bacterial infections was detected. The results of our study showed that this essential oil also has an overall killing activity in mouse macrophage cells. While the MIC value of ciprofloxacin was 0.031 µg/mL with a zone value of 9 ± 0.5 mm, the most successful group in the combination was Penicillin + G.O, with a MIC value of 0.125 µg/mL, and a MIC zone diameter of 11 ± 0.5.

This research provides comprehensive information that can form a scientific basis for the use of Geranium oil in combination with antibiotics in the clinical management of some infectious diseases caused by *S. pneumoniae*, especially upper respiratory tract infections.

## Figures and Tables

**Table 1:** Chemical composition of Geranium oil.

RRI	Compound	%
1032	α-Pinene	0.3
1174	Myrcene	0.1
1176	α-Phellandrene	0.1
1203	Limonene	0.2
1213	1,8-Cineol	0.1
1218	β-Phellandrene	tr
1255	γ-Terpinene	0.1
1280	p-Cymene	0.1
1290	Terpinolene	tr
1362	cis-Rose oxide	1.3
1377	trans-Rose oxide	0.5
1450	trans-Linalool oxide (Furanoid)	0.1
1475	Menthone	2.8
1503	Isomenthone	4.1
1528	α-Bourbonene	tr
1535	β-Bourbonene	0.9
1553	Linalool	2.8
1596	α-Guaiene	0.5
1612	β-Caryophyllene	1.2
1617	6,9-Guaidiene	5.5
1628	Citronellyl formate	9.1
1638	Menthol	0.6
1628	Aromadendrene	0.1
1661	Alloaromadendrene	0.3
1668	Citronellyl acetate	0.4
1677	epi-Zonarene	0.1
1687	α-Humulene	0.1
1706	α-Terpineol	0.3
1715	Geranyl formate	0.9
1708	Ledene	1.6
1726	Germacrene D	0.9
1729	Citronellyl propionate	1.1
1740	α-Muurolene	0.3
1755	Bicyclogermacrene	0.3
1772	Citronellol	41.2
1773	δ-Cadinene	0.4
1776	γ-Cadinene	0.1
1808	Nerol	0.1
1809	Citronellyl butyrate	1.6
1857	Geraniol	8.0
1849	Calamelene	0.1
1889	Myrtanol	0.3
1901	Geranyl butyrate	1.0
1953	Citronellyl 4-methyl valerate	tr
2020	(E)-Citronellyl tiglate	1.1
2080	1,10-di-epi-cubenol	0.2
2122	Neryl tiglate	1.4
2157	Geranyl tiglate	0.1
2214	Phenyl ethyl tiglate	0.8
	Total	93.2

RRI Relative retention indices calculated against n-alkanes; % calculated from FID data; tr Trace (<0.1%).

**Table 2:** MIC zone values of ciprofloxacin and ciprofloxacin + Geranium oil combinations against *S. pneumoniae*.

Ciprofloxacin (µg/mL)	Zone Diameter (mm)	*Geranium* Oil (mg/mL)	Zone Diameter (mm)	* Ciprofloxacin + G.O	Zone Diameter (mm)
1	22.5 ± 1.5	40	15.5 ± 0.5	0.125	21 ± 1
0.5	19 ± 1	20	14 ± 1.5	0.062	17.5 ± 1.5
0.25	16.5 ± 1.5	10	11 ± 0.5	0.031	15 ± 0.5
0.125	15 ± 0.5	5	-	0.015	8 ± 0.5
0.062	13 ± 1	2.5	-	0.007	-
0.031	9 ± 0.5	1.25	-	0.003	-
0.015	-	0.62	-	0.001	-
0.007	-	0.31	-	0.0005	-

* The concentration value of the antibiotic in the combination of ciprofloxacin + G.O is given. (*n* = 3, G.O: *Geranium* oil, data are expressed as mean ± standard deviation).

**Table 3:** MIC zone values of antibiotics and penicillin + Geranium oil combinations against *S. pneumoniae*.

Penicillin (µg/mL)	Zone Diameter (mm)	*Geranium* Oil (mg/mL)	Zone Diameter (mm)	* Penicillin + G.O	Zone Diameter (mm)
16	22 ± 1	40	15.5 ± 0.5	4	27 ± 0.5
8	20.5 ± 0.5	20	14 ± 1.5	2	21 ± 1.5
4	18.5 ± 1.5	10	11 ± 0.5	1	18 ± 0
2	15. ± 0.5	5	-	0.5	14 ± 0
1	16 ± 0	2.5	-	0.25	12 ± 0.5
0.5	13 ± 1	1.25	-	0.125	11 ± 0.5
0.25	-	0.62	-	0.06	-
0.125	-	0.31	-	0.03	-

* The concentration value of the antibiotic in the combination of penicillin + G.O is given. (*n* = 3, G.O: *Geranium* oil, data are expressed as mean ± standard deviation).

**Table 4:** MIC zone values of antibiotics and ampicillin + Geranium oil combinations against *S. pneumoniae*.

Ampicillin (µg/mL)	Zone Diameter (mm)	*Geranium* Oil (mg/mL)	Zone Diameter (mm)	* Ampicillin + G.O	Zone Diameter (mm)
16	26 ± 0.5	40	15.5 ± 0.5	4	20 ± 1
8	24.5 ± 1.5	20	14 ± 1.5	2	18.5 ± 1.5
4	20 ± 0	10	11 ± 0.5	1	17 ± 0
2	18.5 ± 0.5	5	-	0.5	13 ± 0.5
1	16 ± 0.5	2.5	-	0.25	11 ± 1
0.5	12 ± 1	1.25	-	0.125	-
0.25	-	0.62	-	0.06	-
0.125	-	0.31	-	0.03	-

* The concentration value of the antibiotic in the combination of ampicillin + G.O is given. (*n* = 3, G.O: Geranium oil, data are expressed as mean ± standard deviation).

**Table 5:** The overall killing effect of macrophages on *S. pneumoniae* exposed to antibiotics.

*S. pneumoniae* Incubated with Macrophages and Time (Hours)	Control (Log CFU/mL)	Ciprofloxacin (Log CFU/mL)	Penicillin (Log CFU/mL)	Ampicillin (Log CFU/mL)
0	7.04 ± 0.3	6.88 ± 0.10	5.98 ± 0.12	6.88 ± 0.10
2	6.99 ± 0.1	6.30 ± 0.96	4.34 ± 1.89	6.38 ± 0.53
4	6.99 ± 0.0	2.69 ± 0.18	2.76 ± 0.17	2.92 ± 0.40
8	2.75 ± 0.2	2.23 ± 0.36	2.48 ± 0.06	2.63 ± 0.26
12	1.95 ± 0.2	1.78 ± 0.00	1.81 ± 0.00	1.68 ± 0.00

*n* = 3, data are expressed as mean ± standard deviation.

**Table 6:** The overall killing effect of macrophages on *S. pneumoniae* exposed to G.O and G.O + antibiotic combinations.

*S. pneumoniae* Incubated with Macrophages and Time (Hours)	G.O (Log CFU/mL)	Ciprofloxacin + G.O (Log CFU/mL)	Penicillin + G.O (Log CFU/mL)	Ampicillin + G.O (Log CFU/mL)
0	6.04 ± 0.9	6.88 ± 0.10	6.28 ± 0.10	5.19 ± 0.00
2	3.66 ± 0.3	5.96 ± 0.81	4.08 ± 1.36	2.74 ± 0.30
4	2.83 ± 0.1	2.50 ± 0.34	2.12 ± 0.24	2.54 ± 0.18
8	2.05 ± 0.3	1.86 ± 0.07	1.48 ± 0.12	2.32 ± 0.20
12	1.55 ± 0.3	1.60 ± 0.0	1.01 ± 0.03	1.40 ± 0.20

*n* = 3, G.O: Geranium oil, data are expressed as mean ± standard deviation.

## Data Availability

Raw data of this study are available upon request from the corre- sponding author.
